# Thromboembolic Prophylaxis in Total Joint Arthroplasty

**DOI:** 10.1155/2012/837896

**Published:** 2012-09-16

**Authors:** David Knesek, Todd C. Peterson, David C. Markel

**Affiliations:** ^1^Department of Orthopaedic Surgery, Providence Hospital and Medical Centers, 22250 Providence Drive, Suite No. 401, Southfield, MI 48075, USA; ^2^Detroit Medical Center/Providence Hospital Orthopaedic Residency Program, Warren, MI 48092, USA

## Abstract

Approximately 775,000 hip and knee arthroplasties are performed yearly in the United States, with a dramatic increase expected. Patients having hip and knee arthroplasties are at high risk of developing a venous thromboembolism. The American College of Chest Physicians (ACCP) and the American Academy of Orthopedic Surgeons (AAOS) have updated guidelines, which outline new prophylactic strategies. Factor Xa inhibitor rivaroxaban has a new recommendation by ACCP and is gradually being adopted by the joint arthroplasty community as an effective oral agent. Other more well-known agents including warfarin, low-molecular-weight heparin, aspirin, and fondaparinux continue to be options for prophylaxis. While the goal of prophylaxis continues to be the prevention of venous thromboemboli and pulmonary emboli, it is important to consider the increased bleeding risk associated with their use. The most recent ACCP and AAOS guidelines give clinicians a greater autonomy in choosing a prophylactic agent with greater emphasis placed on dialogue between the surgeon and patient as to the choice of prophylaxis.

## 1. Introduction

Approximately 775,000 hip and knee arthroplasties are currently performed yearly in the United States [1]. With the population of adults over 65 years of age in the United States projected to double from 35 million in 2000 to 72 million in 2030 [2], a dramatic increase in the number of total joint arthroplasties (TJAs) will likely be seen. Patients undergoing lower extremity surgery, especially TJA procedures, are inherently at high risk of developing a venous thromboembolism (VTE). Historically, it has been reported that up to 40–60% of patients undergoing total hip (THA) and total knee arthroplasty (TKA) in the absence of thromboprophylaxis will develop either venographic evidence of a deep venous thrombosis (DVT) or a pulmonary embolism (PE) postoperatively [3]. Currently with modern techniques and postoperative care, the estimated risk of developing a symptomatic VTE without prophylaxis is around 4.3% [4].

To address the high patient morbidity and mortality due to VTE, thromboprophylaxis is routinely applied to TJA. Multiple agents, including low-molecular-weight heparin, aspirin, warfarin, fondaparinux, rivaroxaban, and mechanical prophylaxis, have contributed to a reduction in frequency of VTE, although the risk of symptomatic VTE remains approximately 2% within 35 days after major orthopedic surgery [4]. Interestingly, fatal PE has remained consistent in primary THA and TKA, between 0.1 and 2%, no matter which agents are used [5, 6].

Both the American College of Chest Physicians (ACCP) and the American Academy of Orthopedic Surgeons (AAOS) have recognized the risk reduction from antithrombotic agents, but there is still controversy as to which agent(s) has the most efficacy, the appropriate timing of dosing, and the duration of prophylaxis. A delicate balance exists between VTE prophylaxis and systemic and surgical site bleeding, which can lead to surgical wound complications including infection, hematoma, reoperation, and systemic bleeding (gastrointestinal). A large meta-analysis performed by Muntz et al. [7] compared the relative risks of bleeding among the major prophylactic agents (warfarin, heparin, low molecular weight heparin, and pentasaccharides) and determined the extra cost to treat complications associated with bleeds, including reoperation, was $113 per patient receiving prophylaxis; thus, fostering a debate between surgical and medical practitioners as to the risk benefit ratios of different treatment modalities. The purpose of this review is to discuss the efficacy of the most commonly used VTE agents and provide clinicians with treatment options for discussion with their patients.

## 2. Pathogenesis

Rudolph Virchow proposed that 3 etiologic factors give rise to thrombosis: vascular endothelial damage, stasis of blood flow, and hypercoagulability of blood [8]. Risk for VTE increases as predisposing factors increase (Box 1). TJA exposes a patient to each portion of the triad during the operative period. Endothelial damage may occur with retractor placement, dislocation procedures, thermal injury during cement hardening, placement of the prostheses, and lower extremity manipulation during the surgery. Stasis may occur from positioning during the operative procedure, from perioperative swelling, occlusive dressings, and decreased mobility [6]. A hypercoagulable state may occur as a result of blood loss, loss of anticlotting factors, and potentially from lipids and collagen release during the surgical procedure.

Previously published guidelines have advocated for risk stratification of patient factors, but no consensus has been reached as to how to do so or if this stratification is actually of clinical benefit. Some risk factors for VTE include prior VTE, advanced age (>40 years), obesity, cancer history, bed rest >5 days, congestive heart failure, varicose veins, estrogen treatment, stroke, multiple trauma, childbirth, myocardial infarction, and hypercoagulable states such as protein C or S deficiency, antithrombin III deficiency, lupus anticoagulant, antiphospholipid antibodies, and myeloproliferative disorders.

## 3. ACCP Guidelines

The ACCP first published VTE prophylaxis guidelines in 1986 and have subsequently updated their guidelines with the advent of new pharmacologic agents, improved surgical technique, and newer publications. The current 2012 guidelines discuss the “use of prophylaxis to reduce the patient-important outcomes of fatal and symptomatic PE and symptomatic DVT balanced against the hazard of an increase in symptomatic bleeding events.” [4] Recommendations were based on a grading scale of published evidence-based medicine criteria. Grade I indicated strong evidence where benefits did or did not outweigh risk, burden, and cost. Grade II indicated less empirical consensus. Further stratification within each grade included (A) randomized controlled trials, unbiased, consistent results (B) randomized controlled trials with inconsistent results or design flaw, and (C) observational studies. The guidelines are meant as a “guide” to help clinicians sort through the myriad of data for appropriate patient treatments.

The current guidelines (9th edition) for patients undergoing elective THA or TKA include Grade IB evidence for prophylaxis with low-molecular-weight heparin (LMWH), fondaparinux, apixaban, dabigatran, rivaroxaban, low-dose unfractionated heparin (LDUH), adjusted dose vitamin K antagonists (VKA), and aspirin. Specific dosing is not recommended although many of the cited studies do provide specific dosing. There is a Grade 1C recommendation for use of intermittent pneumatic compression devices (IPCDs). These agents are recommended for a minimum of 10–14 days. A Grade 1B recommendation was made to start LMWH >12 hours preoperatively or postoperatively. This followed a systematic review that showed dosing within 12 hours of surgery caused an increase in bleeding. A Grade 2B recommendation was given in favor of LMWH relative to the other agents except VKA or aspirin, which carried Grade 2C. This was in spite of the use of IPCD or treatment duration. There is also a Grade 2B recommendation to extend any VTE treatment up to 35 days from the procedure rather than 10–14 days. Grade 2C evidence suggests dual prophylaxis with IPCDs and a pharmacologic agent while in the hospital, use of IPCDs or no prophylaxis if there is an increased bleeding risk, use of an oral agent other than aspirin if a patient is unwilling to administer injections or use an IPCD, and against the use of IVC filters for primary prevention over no prophylaxis in patients with an increased bleeding risk or contraindication to other prophylaxis. Finally, Grade 1B evidence recommends against screening asymptomatic patients before hospital discharge.

These guidelines differed significantly from previous guidelines. First, several other agents are now mentioned in the recommendations such as aspirin (previously recommended against as a sole agent), dabigatran, apixaban, rivaroxaban, and LDUH (previously recommended against as a sole agent). Second, no specific dosages are recommended (although some are inferred based on cited studies). Third, 2012 recommendations are given for both THA and TKA, not separately as in 2008.

## 4. AAOS Guidelines

The AAOS guidelines [9] consisted of graded levels of evidence from which the authors provided a final recommendation. These were strong (good-quality evidence), moderate (fair-quality evidence), weak (poor-quality evidence), inconclusive (insufficient or conflicting evidence), or consensus (in the absence of reliable evidence, the workgroup made recommendations based on clinical judgment).

The set of ten recommendations were as follows: (1) recommendation against postoperative ultrasound screening (strong); (2) determination whether patient had a previous VTE (weak), unable to recommend for or against assessing patients for VTE risk factors (inconclusive); (3) patients should be assessed for bleeding disorders such as hemophilia or active liver disease (consensus), and unable to recommend for or against using other risk factors to assess bleeding risk (inconclusive); (4) patients should discontinue the use of antiplatelet agents before undergoing elective hip or knee arthroplasty (moderate); (5) the academy suggests the use of pharmacologic and/or mechanical prophylaxis in patients who are not at elevated risk for VTE or bleeding beyond that of the surgery itself (moderate), is unable to recommend for or against specific prophylactic strategies (inconclusive), and in the absence of reliable evidence as to the length of prophylaxis, physicians should discuss the length of prophylaxis with their patients (consensus); (6) the academy recommends that patients with known bleeding disorders or active liver disease use mechanical prophylaxis (consensus); (7) the academy suggests that patients with previous VTE receive pharmacologic and mechanical prophylaxis (consensus); (8) the academy recommends patients should undergo early mobilization (consensus); (9) the academy suggests the use of neuraxial anesthesia to limit blood loss even though its does not affect occurrence of VTE (moderate); (10) the academy is unable to recommend for or against IVC filters (inconclusive).

## 5. Treatment Options

### 5.1. Aspirin

Aspirin (acetylsalicylic acid, ASA) irreversibly blocks cyclooxygenase and the formation of thromboxane A2, a potent platelet aggregator. This results in disabling the clotting ability of platelets (Figure 2). ASA is a very attractive treatment option for many surgeons because of its ease of administration, low expense, decreased risk for bleeding complications, and lack of monitoring. Several studies have shown an added benefit by decreasing the risk of heterotopic ossification [10, 11]. Previous recent studies advocated dosages in a wide range but currently low-dose aspirin (75 mg–325 mg) has been advocated.

The use of aspirin as a VTE prophylactic modality has been a topic of debate for many years. The ACCP has previously advocated against its use alone as a chemoprophylactic agent because several studies have shown other agents to be more efficacious. The ACCP discounted the Antiplatelet Trialist Collaboration [12], which showed that ASA has benefit in protecting against DVT and an even greater benefit in protecting against PE. The ACCP stated that this meta-analysis had a pool of poor quality data, and the DVT detection methods were substandard. They also stated that a number of trials showed no significant benefit and/or inferior results compared to other prophylactic agents. Recently, Woller et al. [13] prospectively studied 696 patients having either TKA or THA and classified 281 as AAOS standard risk or elevated risk (2008 guidelines). The other 415 patients received ACCP-recommended treatment with warfarin or enoxaparin. The standard risk patients receiving ASA had a significantly higher rate of symptomatic PE (4.6% versus 0.7%, *P* < .030) and VTE (7.9% versus 1.2%, *P* < .001) than the patients receiving warfarin. However, in their most current guidelines, the ACCP has given ASA a grade 1B recommendation of support. Current ACCP recommendations state that ASA has a modest risk reduction in symptomatic DVT and that taking low-dose ASA for 35 days will result in seven fewer symptomatic VTEs per 1000 patients. They do caution that ASA may also result in more major nonfatal bleeding episodes compared to placebo (RR 1.12), but there was no difference in bleeding requiring reoperation or bleeding death. There was also a trend toward nonfatal myocardial infarctions compared to placebo (RR 1.59), which resulted in 2 more nonfatal myocardial infarctions per 1000 patients [4].

The AAOS, on the other hand, has advocated for the use of ASA for patients with a standard risk (no history of malignancy, no previous clots, no history of thrombophilia) of PE. Their previous guidelines pointed out simply that if a patient developed a DVT, he or she would not inherently develop a symptomatic DVT or PE. Bozic et al. [14] retrospectively analyzed over 93,000 patients in the United States having primary TKA between October 2003 and September 2005 and found that those patients who received ASA had lower adjusted odds radio for VTE and PE compared to warfarin (OR 1.36, *P* < .01) and similar adjusted odds ratio compared to injectable agents (OR 1.03, *P* < .01). Similarly, in a retrospective analysis of the total joint registry, Khatod et al. [15] showed that treatment with ASA in THA had no difference in the PE, fatal PE, or death compared with other prophylactic measures. Lotke et al. [16] also demonstrated that ASA, combined with other AAOS recommended measures and improved surgical techniques, is safer than and equally efficacious as other chemoprophylaxis agents at preventing fatal PE .06%–0.14% (expected prevalence in literature 0.1%), nonfatal PE 0.26% (expected prevalence in literature <1%), and symptomatic DVT 0.2% (expected prevalence <3%). Currently the 2011 AAOS does not recommend for or against the use of aspirin but simply states that a chemoprophylactic agent should be used.

### 5.2. Rivaroxaban

**Figure 1:**
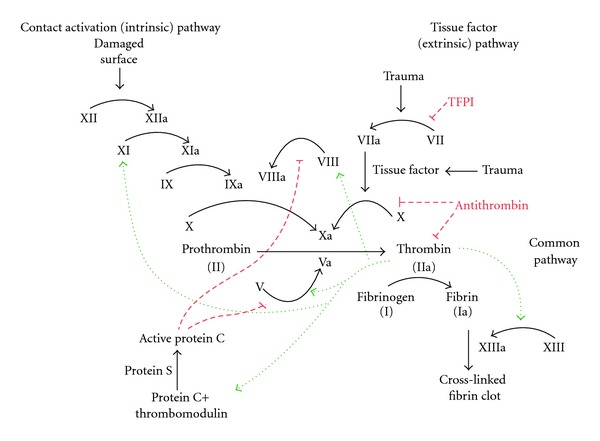
Coagulation Cascade.

**Figure 2:**
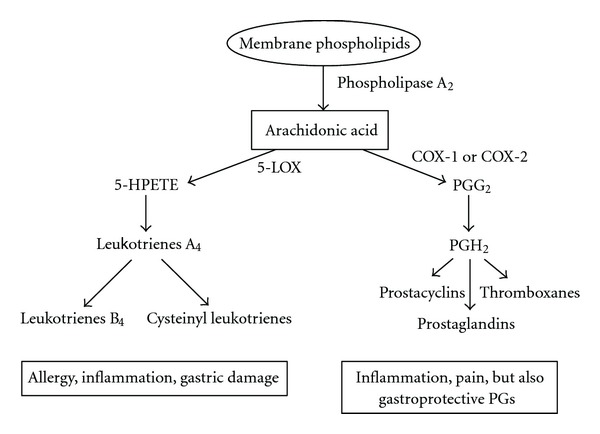
COX pathway.

Rivaroxaban is an oral, once a day, reversible, direct factor Xa inhibitor (Figure 1). It requires no monitoring and has recently been approved for use in the United States for the prophylaxis of VTE in primary THA and TKA. This drug was studied in the RECORD trials (1–4), which evaluated the drug's efficacy against enoxaparin [17]. Almost 13,000 patients were evaluated for primary endpoints of symptomatic DVT, non-fatal PE, and all-cause mortality in both THA (RECORD 1 and 2) and TKA (RECORD 3 and 4) at the end of the medication period. RECORD 1 compared rivaroxaban 10 mg qday for 5 weeks versus 40 mg enoxaparin qday for 5 weeks, and RECORD 2 compared rivaroxaban 10 mg qday for 5 weeks versus 40 mg enoxaparin for 10–14 days plus oral placebo. RECORD 3 compared rivaroxaban 10 mg qday for 10–14 days versus enoxaparin 40 mg qday for 10–14 days, and RECORD 4 compared rivaroxaban 10 mg qday for 10–14 days versus enoxaparin 30 mg BID for 10–14 days. The primary safety endpoints were major bleeding which included surgical site bleeding that required reoperation, major and clinically relevant nonmajor bleeding, and any bleeding. A pooled analysis of these studies showed that rivaroxaban had superiority over enoxaparin in preventing major venous thromboembolism defined as a composite of proximal deep vein thrombosis, nonfatal pulmonary embolism, or death from venous thromboembolism [RECORD 1–3 (0.5% versus 1.3%  *P* < .001)] and RECORD 4 (6.9% versus 10.1%  *P* < .0118)]. With regards to safety endpoints, there was no statistical significance between enoxaparin and rivaroxaban in any RECORD studies. In a similar positive analysis, Kwong [18] showed that rivaroxaban was a cost-effective alternative to enoxaparin.

Most surgeons are concerned about the potential for surgical site bleeding and hematoma formation. These complications may lead to drainage, infection, and return to the operative suite. This is associated with patient morbidity and a significant cost burden. In a retrospective cohort study, Jensen et al. [19] evaluated this issue in 1048 patients having THA and TKA. One group of 489 patients received tinzaparin 4500 u qday, and one group of 536 patients received rivaroxaban 10 mg qday. In the first 30 days post-op, 9 (1.8%) patients in the tinzaparin group returned to the operative suite for open irrigation and debridement for either infection or hematoma, compared to 22 (3.94%) patients in the rivaroxaban group. This increase was statistically significant, *P* = .046.

While rivaroxaban has shown prophylactic efficacy in both THA and TKA, it must be used with caution as it may cause an increase in surgical site complications. Its ease of use as an oral agent that requires no monitoring may also be advantageous in noncompliant patients.

### 5.3. Warfarin

Discovered in 1921 and used as a rodenticide since the 1940s, warfarin has been used as low cost anticoagulant since the 1950s. Warfarin impairs the creation of the clotting factors II (prothrombin), VII, IX, and X by inhibiting vitamin K epoxide reductase (Figure 1). Multiple meta-analyses and trials have compared warfarin to LMWH, ASA, LDUH, and mechanical prophylaxis and have showed its efficacy. With compliance of INR 2-3, warfarin appears similar in VTE prevention and has lower rates of bleeding complications compared to LMWH and LDUH [13, 20, 21].

Unfortunately, it may be difficult to control the therapeutic window of warfarin. In a retrospective cohort design, Nordstrom et al. [22] evaluated INR patterns in a cohort of patients who had received a TKA/THA relative to VTE prevalence. They found that the majority (~83%) of THA and TKA patients were not therapeutic (INR 2-3) on post-op day 4 of their surgery, a time of increased VTE risk. Over the duration of their warfarin treatment (32–35 days), roughly one-third of the INR levels (33.3% THA and 28.6% TKA) fell within the 2008 ACCP-recommended INR range of 2.0–3.0. Moreover, in both cohorts of patients, VTE was strongly dependent on the INR with a 4-5-fold increased risk for VTE in patients who did not achieve an INR >2.0.

Drug interactions and bleeding risks with warfarin are of concern. Warfarin is metabolized by the cytochrome P450 enzymes and has a long list of drug interactions, some of which enhance (e.g., quinolones, trimethoprim/sulfamethoxazole, and antifungals) and that diminish (e.g., phenytoin, carbamazapine, phenobarbital, and rifampin) the efficacy of its anticoagulation. This property makes dosing and maintaining a consistent INR value very difficult. Delayed onset of action also may make management difficultly, leading many physicians to bridge this “gap” of sub-therapeutic INR with another chemoprophylaxis (e.g., UFH or LMWH). Large loading doses, elderly persons >75, liver disease, and drug interactions are all risk factors for increasing INR and increasing bleeding. Although warfarin has excellent efficacy within its therapeutic window, difficulties remain dealing with early and late INR variations as well as the albatross of chronic monitoring.

### 5.4. LMWH

LMWHs bind to and accelerate the activity of antithrombin III, potentiating the inhibition of coagulation factor Xa and thrombin (Figure 1). LMWH has more controlled binding properties to plasma proteins than unfractionated heparin, delivering a more consistent dose response, better bioavailability, and less potential to cause heparin-induced thrombocytopenia (HIT) [23]. The combination of its consistent bioavailability, linear pharmacokinetics, and short half-life make it a very attractive option for the prevention of VTE.

Many trials have compared the efficacy of LMWH to warfarin and UFH in the prevention of DVT and PE following joint arthroplasty. Many prospective, randomized, and sometimes multi-institutional trials have found LMWH to be superior to UFH and warfarin in the prevention of DVT [24–26]. Fitzgerald et al. [25] performed a randomized, multicenter trial of 349 patients comparing enoxaparin (30 mg SC BID) to warfarin (INR 2-3) and found an estimated odds ratio for the development of VTE to be 2.52 times greater with warfarin than enoxaparin. Although not statistically significant, the episodes of major and minor hemorrhagic episodes in the warfarin treated patients (23%, 41 of 176) were lower than that of the enoxaparin treated group (34%, 58 of 173). In a prospective randomized trial, Warkentin et al. [23] had similar results with a significantly lower rate of DVT in the LMWH group compared to warfarin (15% compared to 26%, *P* = .006). Blood loss and decreased hematocrit between the two groups were similar; however, the LMWH group required more blood transfusions in the 1st through 8th postoperative day and, as mentioned in the aforementioned pro-warfarin studies, had more operative site bleeding complications.

Postoperative complications following early prophylaxis with LMWH are well outlined in the literature and include wound necrosis, sciatic nerve palsy secondary to postoperative hematoma, drainage, and persistent hematoma at the surgical site. Due to its rapid onset of action, it should not be initiated for 12 hours after neuraxial anesthesia in order to prevent epidural hematoma and neurologic deficits, and it is contraindicated in individuals with indwelling spinal catheters. LMWH should not be administered for at least 4 hours after an epidural catheter has been removed. All of these factors play a crucial role in balancing the benefits of DVT/PE prevention versus the deleterious complications associated with bleeding.

### 5.5. Fondaparinux

Fondaparinux is a synthetic oligosaccharide that is based on the pentasaccharide sequence of the heparin and LMWH molecules that is responsible for its binding affinity to antithrombin III (Figure 1). This selective affinity allows for less interaction with plasma proteins and other molecules in the clotting cascade. Fondaparinux has been proven safe in the treatment of patients with HIT, demonstrating no HIT-like reaction in these patients' serum samples. Being almost exclusively excreted by the kidneys, it must be used judiciously for patients with renal disease having a creatinine clearance <50 mL/min [27, 28], and it is contraindicated in patients with CrCl <30.

The majority of fondaparinux trials for prevention of VTE after arthroplasty have been in direct comparison to LMWH due to their similarity. A meta-analysis of 4 randomized double-blinded studies found that fondaparinux (2.5 mg SC daily) administered 6 hours after major orthopedic surgery significantly reduced the incidence of VTE by day 11 [182 (6.8%) of 2682] compared with enoxaparin administered either 40 mg SC daily or 30 mg SC twice daily [371 (13.7%) of 2703], *P* < .01 [29]. While the rate of VTE was lower, there was no difference in the reduction of fatal PE, and importantly there was a statistically significant increase in major bleeding episodes in the fondaparinux group [96 (2.7%) of 3616] compared to the enoxaparin group [63 (1.7%) of 3621], *P* = .008. However, there was no difference in the incidence of clinically relevant bleeding between the two groups leading to death or reoperation. Bauer et al. [30] confirmed these data with a prospective, randomized, double-blind study of 1049 patients undergoing elective TKA. They found a statistically significant decrease in VTE by day 11 in the fondaparinux compared to enoxaparin (12.5% to 27.8%) with a risk reduction of 55.2% (*P* < .001). Similarly, major bleeding episodes occurred more frequently in the fondaparinux group. Turpie et al. [28] examined the relationship of bleeding and the timing of the first dose of fondaparinux in patients undergoing elective major hip and knee surgery, or standard surgery for the fracture of the proximal third of the femur. When fondaparinux was given 3–6 hours of surgery, the incidence of major bleeding was higher (3.2%, 42/1300) than when it was given within 6–9 hours of surgery (2.1%, 46/2171, *P* < .045); although when factoring out major bleeds that occurred before administration of the first dose of fondaparinux (15/88), the difference between the 2 groups does not reach statistical significance, *P* < 0.18. Thus, there is still a debate about the timing of the first postoperative dose of fondaparinux with most clinicians recommending at least 6–8 hours postoperatively before administration.

Fondaparinux appears to have excellent efficacy in the prevention of VTE following major orthopedic surgery but shows no superiority in the prevention of fatal PE. However, it carries a significantly higher risk of major bleeding episodes compared to LMWH. Clinical judgment must be used in patients with renal impairment, which is common in elderly patients undergoing TJA, and patients weighing <50 kg. Therefore, while effective, this medication should be used with caution if bleeding is of concern.

## 6. Discussion

The newer AAOS and ACCP guidelines seem to be approaching the center with their views on prophylaxis. The previous 2008 ACCP guidelines were more specific with what they deemed necessary for full prophylaxis against DVT, gave dosages, INR ranges, and timelines within which to start and stop prophylaxis. They also did not appear to appreciate operative site bleeding as much as many surgeons would like to. The new guidelines are more accepting of varied pharmacologic prophylaxis and definitions of adequacy. The current guidelines recommend aspirin as a choice for prophylaxis and are accepting of some new factor Xa and thrombin inhibitors. In addition, they have shifted focus away from asymptomatic DVT and other clinically irrelevant end points to the more relevant symptomatic DVT and PE. This is a change that has been advocated by the AAOS.

The AAOS has also shifted its focus from past years' guidelines. Unlike previous years, the 2011 guidelines were unable to conclude on any specific prophylactic strategies and were also unable to make recommendation regarding the length of postoperative prophylaxis. They allow the surgeon more autonomy based on clinical judgment for the prevention of postoperative VTE. The AAOS encourages active discussion between patient and surgeon regarding the risks and benefits of the specific treatment agents as well as the length of postoperative prophylaxis. Some surgeons may disagree with this strategy for medical, legal or other reasons and may desire more standardized care. However, the goal appears to be engagement of the patient in the clinical decision-making and a focus on individualized care recommendations.

## 7. Conclusions

More prophylactic options exist now than with previous guidelines. New to treatment guidelines are apixaban, dabigatran, and rivaroxaban, all of which are recommended by the ACCP for prophylaxis (only rivaroxaban is approved for use in the United States). These agents seem to have excellent efficacy in preventing VTE but may increase operative site bleeding. Fondaparinux is also a very efficacious choice but operative site bleeding seems to continue to be a concern. Enoxaparin has remained a stalwart in the treatment of VTE and may now be seen as the gold standard for this class of agent, keeping bleeding complications relatively low while remaining very efficacious. The new guidelines have put aspirin in a new light. A shift from previous practice has placed aspirin on par with other prophylactic strategies. Warfarin remains a steady choice for many orthopedic surgeons. Its efficacy cannot be questioned, but the bleeding risk and the cumbersome monitoring make its use problematic.

Many prophylactic choices are available to orthopedic surgeons, all of which have demonstrated efficacy. Unfortunately with this efficacy comes the constant concern of bleeding risks. New guidelines give surgeons increased freedom to decide which agent provides their patients an acceptable bleeding risk and reduction in VTE risk. This dialogue will become an integral part of the surgeon-patient relationship as more and more people have joint replacement surgery.

## Figures and Tables

**Box 1:**
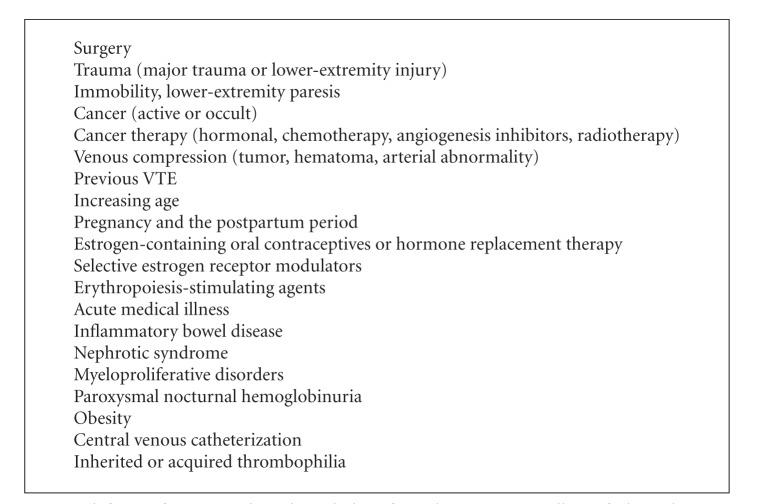
Risk factors for venous thromboembolism from the American College of Chest Physicians.
